# Assessment of the RoAD Score as a Predictor of Long-Term Irreversible Ambulatory Disability in Multiple Sclerosis: Evidence from a Real-World Cohort

**DOI:** 10.3390/medicina61122121

**Published:** 2025-11-28

**Authors:** Tommaso Guerra, Chiara Esposto, Sara Limitone, Damiano Paolicelli, Pietro Iaffaldano

**Affiliations:** Department of Translational Biomedicine and Neurosciences-DiBraiN, University of Bari “Aldo Moro”, 70100 Bari, Italy; chiaraesposto97@gmail.com (C.E.); limisara@gmail.com (S.L.); damiano.paolicelli@uniba.it (D.P.)

**Keywords:** multiple sclerosis, risk score, prognosis

## Abstract

*Background and Objectives*: In multiple sclerosis (MS), the Risk of Ambulatory Disability (RoAD) score is a validated prognostic tool based on demographic, clinical, and MRI variables assessed at treatment initiation and after one year. We aimed to assess the predictive value of the RoAD score for long-term irreversible disability in a large real-world MS cohort. *Materials and Methods*: A retrospective analysis was performed on relapsing–remitting MS patients (RRMS) followed for ≥5 years at the Bari MS Center. The RoAD score (0–8) was calculated and dichotomized (<4 vs. ≥4). Cox proportional hazards models were used to evaluate the risk of irreversible Expanded Disability Status Scale (EDSS) 6.0. *Results*: A total of 1051 RRMS patients (67.1% female) were included, with a median follow-up of 18.0 years. Most patients (97.5%) received moderate-efficacy DMTs as first-line therapy. During follow-up, 123 (11.7%) patients reached irreversible EDSS 6.0 after a median of 9.5 years. Patients with RoAD score ≥4 (16.8%) showed a threefold higher risk of irreversible disability (HR 3.16, 95% CI 2.19–4.56, *p* < 0.001). The predictive value of RoAD ≥4 was confirmed both in the adjusted multivariable model and in sensitivity analyses treating RoAD as a continuous variable. *Conclusions*: In this real-world cohort, the RoAD score demonstrated solid predictive validity for long-term ambulatory disability, supporting its role as a practical tool for early risk stratification in MS management.

## 1. Introduction

Accurate prognostication is a cornerstone of modern clinical practice, yet it remains a considerable challenge. This concept holds true across general medicine and chronic disease management, as well as in neurology, and is most notably applicable in the field of multiple sclerosis (MS) [1,2,3]. The long-term course of MS is highly variable, and individual outcome prediction is particularly complex. This variability stems from the complex interplay between several immunopathological mechanisms that drive both inflammatory and neurodegenerative phenomena over time [4,5], thereby limiting disease outcome modeling. Therefore, identifying patients at a higher risk of progression remains an unmet clinical need, especially in the context of early therapeutic decision-making. Despite several predictive models for personalized patient management, no validated prognostic score has yet been incorporated into routine clinical practice [6]. The integration of clinical, imaging, and biological data into a unified and reproducible prognostic framework represents a major priority in the MS field.

The Risk of Ambulatory Disability (RoAD) score is a validated prognostic tool useful to predict individual prognosis and guide treatment strategies in relapsing remitting MS (RRMS) patients [7]. This score includes demographic and clinical factors assessed at the initiation of the first disease modifying treatment (DMT)—age, disease duration, Expanded Disability Status Scale (EDSS)—in parallel with predictors of treatment response after one year of treatment—assessment of clinical relapses and radiological worsening at follow-up magnetic resonance imaging (MRI) scans. The score was developed using independent datasets of patients with RRMS initiating interferon beta or glatiramer acetate. The training cohort consisted of patients from three MS centers in Italy, while the validation was conducted in an independent cohort of patients followed in Barcelona, Spain.

Ambulatory impairment represents a crucial barrier to independence, leading most individuals with MS and their caregivers to consider it a substantial issue that adversely affects quality of life [8]. Therefore, the evaluation of the risk of ambulatory disability is crucial for therapeutic decision-making in MS, particularly considering the long-term impact of early treatment choices on functional outcomes [9]. Greater clinical influence of long-term prognosis evaluation is achieved when several biomarker and therapy response measurements are combined with clinical and demographic predictive variables [10]. Early clinical features in MS strongly influence the trajectory of the disease, including the timing of transition to secondary progressive MS (SPMS) and the risk of disability accumulation [11].

A study, conducted in a Swiss university MS clinic, independently evaluated the RoAD score, and confirmed the evidence supporting its utility as a prognostic tool for early prediction of disability progression in patients with RRMS [12]. Given the rapid development and broad availability of modern DMTs, evaluating early prognostic markers is essential to identify patients who may benefit from more intensive treatment strategies.

In this study, we aimed to validate the application of the RoAD score in a large real-world Italian cohort, assessing the performance in prognosticating the irreversible disability accrual, exploring whether this tool could represent a practical step toward a more individualized approach to MS care. This study aims to enhance prognostic debate by examining a tool based on commonly accessible clinical indicators, utilizing a significantly larger cohort than prior research.

## 2. Material and Methods

### 2.1. Data Collection and Study Population

All data on demographics, treatments, and regular neurological evaluations of patients included in this study were collected from the Italian MS Register (RISM) WebApp and clinical records. The RISM study was approved by the Ethics Committee of the “Azienda Ospedaliero-Universitaria Policlinico of Bari”, and patients signed an informed consent that allows research purposes [13].

Data extraction was performed by applying manually sequential filters to the database in May 2025. A minimum dataset was retrieved including the following variables: date of birth, sex, disease onset and MS diagnosis, symptoms at onset, clinical relapses (including dates, relapse characteristics, and treatments administered), neurological evaluations with EDSS (with dates and detailed functional system scores), dates of initiation and discontinuation for the first prescribed DMTs, and cerebrospinal fluid (CSF) oligoclonal IgG bands (OCBs).MRI data, including the number of new or enlarged T2 lesions and the presence of gadolinium-enhancing lesions, were retrieved from the RISM WebApp and complemented with information extracted from the patients’ electronic medical records. These variables were then manually integrated into the study dataset to ensure accurate quantification of radiological activity. Neurological evaluations, including EDSS assessments, were performed on an annual basis, with at least one visit per year. Missing values in the RISM-App were verified and complemented using available clinical records to ensure data completeness.

A retrospective, monocentric analysis was conducted, considering the following inclusion criteria: (1) patients with a RRMS course; (2) at least 5 years of follow-up; (3) treatment with at least one DMT; (4) an initial EDSS < 4.0; (5) annual neurological evaluations; (6) MRI scans with complete data on lesion count at baseline (treatment start) and after one year of therapy. DMT exposure was classified considering the classification in moderate efficacy (ME DMTs: interferon beta products, glatiramer acetate, teriflunomide, dimethyl fumarate) and high efficacy (HE DMTs: natalizumab, fingolimod, ocrelizumab) therapies.

### 2.2. Statistical Analysis

For descriptive analysis, continuous variables have been calculated as median with interquartile ranges or mean and standard deviation (SD), and categorical variables have been presented as frequencies (proportions).

The scoring system of the RoAD index, ranging from 0 to 8, was applied exactly as defined by its original items (7). At the start of initial treatment, points are assigned for age ≥ 40 years (1 point), disease duration ≥ 2 years (1 point), and EDSS between 1.5 and 2.0 (1 point). After one year of treatment, additional points are given for disease activity, namely the occurrence of one relapse (1 point), ≥2 relapses (2 points), the presence of ≥1 gadolinium-enhancing lesion (1 point), and ≥3 new T2 lesions (2 points). The total RoAD score, obtained by summing all items, was calculated for all patients of the cohort according to the parameters reported above. The irreversible EDSS 6.0 achievement was defined by EDSS scores greater than or equal to 6.0 followed by never lower EDSS ratings in all the subsequent follow-up visits. In the ME DMT subgroup, crude proportions of patients reaching irreversible EDSS 6.0 were compared between oral and injectable therapies using Pearson’s chi-square test.

**Figure 1:**
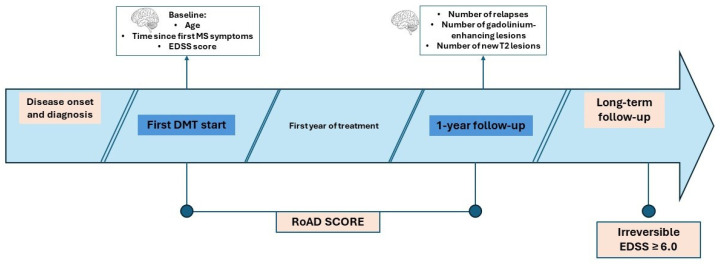
Study timeline. Schematic representation of key timepoints of in the analysis: disease onset and diagnosis, first DMT initiation (baseline for RoAD calculation), and follow-up. Abbreviations: DMT, disease-modifying therapy; EDSS, Expanded Disability Status Scale; RoAD, Risk of Ambulatory Disability.

Figure 1 illustrates the study timeline and the timepoints used to define baseline and follow-up.

In the survival analysis to estimate the risk of reaching irreversible EDSS ≥ 6.0, a Cox regression model was performed by applying the RoAD score dichotomized (<4 vs. ≥4) as the independent variable. Hazard ratios (HR) were displayed with the 95% confidence intervals (95% CI). Time to event was calculated from baseline to the first occurrence of irreversible EDSS ≥ 6.0 from DMT initiation; censoring occurred at the date of the last recorded clinical assessment for patients who had not reached the endpoint.

In the main time-to-event analysis, we also adjusted the Cox models for sex, age at onset, and treatment class. Sex was modeled as a binary variable (female vs. male, male as reference), age at onset was entered as a continuous covariate (per 1-year increase), and treatment class was dichotomized into ME versus HE DMTs (ME as reference).

As a sensitivity analysis, we also modeled the RoAD score as a continuous predictor. A multivariable Cox proportional hazards model was fitted including sex, age at onset, and initial DMT class as covariates to evaluate whether a linear increase in the RoAD score was associated with the hazard of reaching irreversible EDSS 6.0.

To assess potential deviations from linearity in the association between the continuous RoAD score and the hazard of irreversible EDSS 6.0, we fitted an additional multivariable Cox model including both the linear term of RoAD and a second-degree polynomial term (RoAD^2^). This approach is recommended as a standard surrogate of spline functions, allowing testing for non-linear effects of continuous predictors. A non-significant quadratic component was interpreted as absence of relevant deviation from linearity [14,15]. The model was adjusted for sex, age at onset, and initial DMT class.

As an additional exploratory analysis, we restricted the Cox regression to the subgroup of patients treated with ME DMTs only, further stratifying the cohort by route of administration (injectable vs. oral). The multivariable model was adjusted for the same baseline covariates used in the primary analysis (sex and age at onset), and the RoAD score was entered as a dichotomous predictor (≥4 vs. <4).

All statistical analyses were performed in Windows SPSS Statistics Version 31.0.1.0 (IBM Corporation, Armonk, NY, USA). A threshold of 0.05 was used for statistical significance.

## 3. Results

After applying the inclusion criteria, we retrieved a final cohort of 1051 RRMS patients, of whom 705 (67.1%) were female. The baseline demographics and clinical characteristics of the cohort are detailed in Table 1. The mean (SD) age of onset was 28.34 years (9.13), and the time (median, IQR) between diagnosis and initiation of first DMT therapy was 0.36 (0.16–0.99) years, while the mean (SD) age of first treatment start was 32.29 (±9.95). At onset, a median (IQR) EDSS of 2.0 (0–3.5) was detected in the overall cohort. The median (IQR) follow-up duration was 18.01 (12.76–23.01) years and we reported a median (IQR) number of visits per patient of 20 (13–30).

Considering DMT distribution, 1025 (97.52%) subjects were first treated with ME DMTs, including 970 receiving injectable therapies, 863 (84.2%) first treated with interferon-beta products and 107 (10.43%) with glatiramer acetate, and 55 receiving oral agents, considering 41 (4%) receiving dimethyl fumarate and 14 (1.37%) teriflunomide. The number of patients treated with HE DMTs was 26 (2.5%), including 8 treated with natalizumab, 3 with ocrelizumab, and 15 with fingolimod. DMT distribution in the cohort is reported in Table 2.

**Figure 2:**
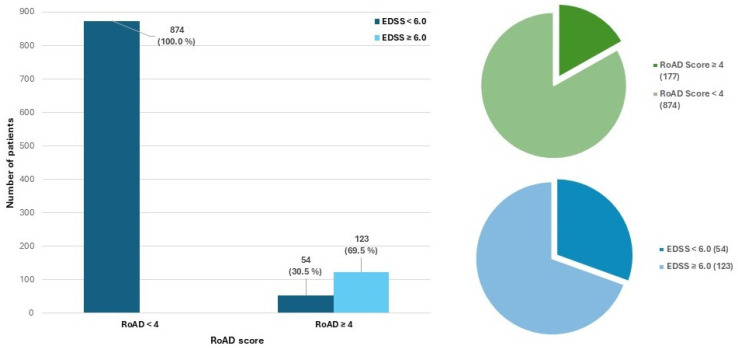
Bar chart and pie charts illustrating the distribution of patients according to RoAD score categories (RoAD < 4 vs. RoAD ≥ 4) and the proportion of subjects who reached irreversible EDSS 6.0 within each group. The bar plot displays the absolute number and percentage of patients reaching (light blue) or not reaching (dark blue) EDSS 6.0 for each RoAD category. The pie charts summarize the overall RoAD score distribution (upper panel) and the proportion of EDSS 6.0 events in the entire cohort (lower panel). Abbreviations: EDSS, Expanded Disability Status Scale; RoAD, Risk of Ambulatory Disability.

A total number of 123 (11.7%) patients reached the disability milestone of irreversible EDSS 6.0 after a median (IQR) time of 9.47 (5.80–14.81) years. In the ME DMT subgroup, the proportion of patients reaching irreversible EDSS 6.0 did not significantly differ between oral and injectable therapies (5.4% vs. 12.1%, χ^2^ = 2.25, *p* = 0.13). The distribution of RoAD categories and the proportion of patients reaching EDSS 6.0 within each group are shown in Figure 2.

**Figure 3:**
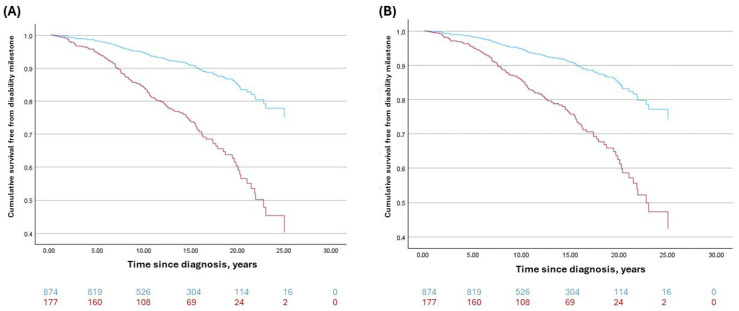
Survival analysis of reaching EDSS ≥ 6.0 based on RoAD score ≥ 4 (red line) and <4 (blue line) by Cox regression analysis. (**A**) Unadjusted regression analysis on RoAD score; (**B**) Regression controlled for sex, age at onset and treatment class (ME vs. HE DMTs). Numbers at risk at each timepoint are reported below the *x*-axis.

When stratified according to the RoAD score threshold of four, 874 patients (83.2%) were classified as having a RoAD score <4, indicating a lower predicted risk of ambulatory disability, whereas 177 patients (16.8%) had a RoAD score ≥ 4, consistent with a higher-risk profile [7]. Considering this stratification, the Cox model demonstrated a HR of 3.16 of reaching an irreversible EDSS ≥ 6.0 (95% CI 2.19–4.56, *p* < 0.001). In the Cox model adjusted for sex, age at onset and treatment class (ME vs. HE DMTs), RoAD ≥ 4 remained strongly associated with the risk of reaching irreversible EDSS 6.0 (adjusted HR 2.89, 95% CI 1.98–4.22; *p* < 0.001). Figure 3 reports the Kaplan–Meier curves comparing the risk of irreversible EDSS 6.0 in patients with RoAD ≥ 4 versus < 4.

The RoAD score was also modeled as a continuous predictor in the Cox analysis, adjusted for sex, age at onset and initial DMT class, retaining a strong and significant association with long-term disability. Each 1-point increase in RoAD corresponded to a markedly higher hazard of reaching irreversible EDSS 6.0 (adjusted HR 1.52, 95% CI 1.30–1.77, *p* < 0.001). This translates into an approximately 52% increase in disability risk for every additional point in the score, supporting the presence of a progressive risk gradient across the entire RoAD range.

To assess whether the association between RoAD and disability risk deviated from linearity, a quadratic term (RoAD^2^) was included in the model. RoAD retained its strong association with the hazard of irreversible EDSS 6.0, while the quadratic component was not significant (*p* = 0.58), suggesting no evidence of non-linear effects. Thus, a linear model for RoAD was deemed appropriate.

In the subgroup restricted to patients treated with ME DMT (n = 1025), the association between RoAD ≥ 4 and irreversible EDSS 6.0 remained strong and highly significant (adjusted HR 3.04, 95% CI 2.08–4.44, *p* < 0.001). No significant difference was observed between oral and injectable ME DMTs (HR 1.24, 95% CI 0.39–3.96, *p* = 0.72).

## 4. Discussion

The RoAD score, combining into a single numerical index clinical and demographic data at treatment initiation and variables related to the effectiveness of therapy at a 1-year time frame, identifies patients who are at higher risk of poor long-term prognosis [7]. This real-world study confirms the clinical utility of the RoAD score as a pragmatic and easily applicable prognostic tool for predicting long-term disability accrual in RRMS patients. In our large single-center cohort of 1051 patients, a RoAD score ≥ 4 was associated with a threefold higher risk of reaching irreversible EDSS 6.0.

Our findings are consistent with the results of the study by Pistor et al. [12], which also focused on the application of the RoAD score to a real-world single-center MS cohort in Switzerland. Patients with RoAD ≥ 4 had a markedly higher risk of EDSS ≥ 4 (HR 3.9, 95% CI 1.6–9.8; *p* < 0.01), confirmed in adjusted Cox models for sex, 1st year immunotherapy and treatment switches. The association with EDSS ≥ 6.0 showed a similar trend (HR 4.3, 95% CI 0.6–30.7) but did not reach statistical significance. While the clinical validity of the RoAD score has been consistently demonstrated in previous cohorts, our study extends this evidence by showing that its predictive performance substantially improves when applied to a larger population with longer follow-up. This reinforces the robustness of the score and highlights its utility in real-world settings characterized by broader and more heterogeneous patient cohorts. Pistor et al. [12] also demonstrated that the cutoff of ≥4 on the RoAD score was associated with a sensitivity of 42.1% (95% CI, 21.1–66.0) and a specificity of 85.4% (95% CI, 78.3–90.5). For this reason, and in line with the cutoff employed in the original validation study, we also adopted a threshold of 4 in our analysis.

Notably, the prognostic effect of RoAD ≥ 4 remained significant after adjustment for sex, age at onset and treatment class (ME vs. HE DMTs), indicating that the score captures an independent component of long-term disability risk beyond demographic and treatment-related confounders. The sensitivity analysis confirmed a progressive and clinically meaningful risk gradient, with each additional point increasing the hazard of irreversible EDSS 6.0 by more than 50%. In addition, the absence of non-linear effects, as indicated by the non-significant quadratic term, suggests that this association is well characterized by a linear trend, reinforcing the interpretability of the score across its range. The subgroup analysis restricted to patients first treated with ME DMT showed that the prognostic effect of the score remained highly consistent, regardless of whether patients’ treatment with oral or injectable agents. Although no significant differences emerged between ME administration modalities, likely due to the small number of patients treated with oral ME and the corresponding wide confidence interval, this finding reinforces the robustness of RoAD across different therapeutic contexts.

Another study compared different scoring systems, including the RoAD score alongside the Rio score, and the modified Magnetic Resonance Imaging in MS (MAGNIMS) score, demonstrating that the RoAD score was a sensitive predictor of progression to EDSS 4.0 and 6.0 among MS patients treated with injectable therapies [16]. Over the past years, multiple prognostic models integrating biomarkers, treatment response metrics, and clinical-demographic features have been validated, offering a more comprehensive and reliable estimation of long-term prognosis compared with single-variable assessments. The Barcelona Risk Score (BRS) is a recent valuable example of a tool aimed to support decision-making in everyday clinical practice by incorporating several biomarkers and by classifying patients into four data-driven groups according to the risk of moderate long-term disability. This score takes into account: relapse-associated worsening phenomena, progression independent on relapse activity events, SPMSconversion, MRI features, and patient-reported scores [17]. The BRS expands prognostic evaluation by integrating emerging concepts of the neuroinflammatory–neurodegenerative continuum in MS. However, despite these broader and newer perspectives, the RoAD score remains a pragmatic and accessible tool, grounded in clinical and MRI parameters that are routinely available to neurologists.

The Bayesian Risk Estimate for MS at Onset (BREMSO) is an individualized prognostic score derived from demographic and clinical variables collected at disease onset. In a large observational cohort from the MSBase registry (N = 14,211), median BREMSO values were significantly higher among patients with greater long-term disease, as measured by the Multiple Sclerosis Severity Score (MSSS), and among those who converted to SPMS [18,19].

A prospective cohort study of newly diagnosed RRMS patients demonstrated that serum neurofilament light chain (sNfL) concentrations combined with optical coherence tomography (OCT) metrics, including retinal nerve fiber layer (RNFL) and ganglion cell–inner plexiform layer (GCL-IPL) thickness, and the clinical score BREMSO, can accurately predict early disability progression assessed with the EDSS-Plus score. In this study, the RoAD score was also evaluated and showed statistically significant differences between EDSS-Plus progressors and non-progressors, alongside RNFL and GCL-IPL thinning [20].

By analyzing a cohort with extended follow-up, our study captures a historical MS population largely exposed to moderately effective DMTs, mainly interferon-beta products, mirroring the training and validation population of the RoAD score [7] and offering a representative picture of patients traditionally treated with therapies of limited immunologic effect as the first approved DMTs. This also allowed the analysis of a cohort in which irreversible disability outcomes are more frequently observed, an aspect that is less represented in populations treated with HE DMTs. High-efficacy therapies have substantially reshaped the therapeutic landscape of MS in recent years [21]. Future studies including cohorts with greater exposure to these treatments will be essential to determine how prognostic scores such as RoAD perform across different treatment eras and to validate their utility in contemporary clinical practice.

Several limitations of this study should be acknowledged. First, this was a single-center retrospective registry-based study and, therefore, data entry errors and inaccuracies cannot be excluded. MRI data evaluation relied solely on real-world lesion counting (new/enlarged T2 and gadolinium-enhancing lesions) rather than harmonized or centralized MRI research protocols, introducing potential variability across radiological evaluations. Moreover, our dataset did not include key neuroimaging biomarkers such as brain atrophy measures, cortical lesions assessment, or metrics of slowly expanding lesions, which are increasingly recognized as important contributors to long-term disability accrual [22,23]. These aspects should be addressed in future studies to refine neuroimaging aspects of prognostic scores. A further limitation is the dichotomization of the RoAD score at the≥4 threshold: the dichotomization of a continuous score could reduce statistical power and underestimate risk gradients across intermediate values. Our sensitivity analyses modeling RoAD as a continuous predictor confirmed the presence of a linear risk gradient, partially mitigating this limitation. Moreover, only a minimal proportion of patients received HE DMTs, limiting comparisons and potentially reflecting treatment-era effects. Our analysis focused on the validation of the RoAD score, considering clinical characteristics at first treatment initiation rather than undertaking a comprehensive evaluation of long-term treatment trajectories. The long median follow-up strengthens the prognostic robustness of the RoAD score, allowing a more reliable assessment of irreversible disability milestones and reducing the risk of short-term fluctuations. Future research should also address the burden of ‘invisible’ MS symptoms, such as fatigue, pain, sleep disturbances, and sphincter dysfunction [24]. These symptoms are highly prevalent even at mild disability levels and show strong associations with overall quality-of-life measures, underscoring their relevance for comprehensive prognostication algorithms.

## 5. Conclusions

The current landscape of MS management has shifted toward a treat-to-target strategy, which requires accurate prognostication to predict trajectories of MS disability [25]. This reinforces the need for integrative algorithms capable of providing a comprehensive assessment by combining biomarkers that capture complementary dimensions of the disease, including clinical presentation and neuroimaging features. In this real-world cohort, we validated the applicability of the RoAD score, which emerged as an accessible and robust tool for predicting disability in MS.

## Figures and Tables

**Table 1:** Baseline clinical and demographic characteristics.

	N = 1051
Female sex, n (%)	705 (67.1)
Age at onset, mean ± SD, years	28.34 ± 9.13
Time between onset and diagnosis, median (IQR), years	1.26 (0.44–4.27)
Time between onset and first DMT start, median (IQR), years	0.36 (0.16–0.99)
Follow up, median (IQR), years	18.01 (12.76–23.01)
Age at first DMT start (baseline of the RoAD score), mean ± SD, years	32.29 ± 9.95
EDSS at onset¸ median (IQR)	2.0 (0–3.5)
Presence of OCBs in CSF, n (%)	967 (92)
Number of OCBs in CSF, median (IQR)	14 (1–34)
Number of visits for patient, median (IQR)	20 (13–30)
Patients initiated with ME DMTs, n (%)	1025 (97.52)

Abbreviations: CSF, cerebrospinal fluid; DMT, disease modifying treatment; EDSS, Expanded Disability Status Scale; HE DMTS, high efficacy DMTs; ME DMTs, moderate efficacy DMTs; MS, multiple sclerosis; OCBs, oligoclonal band.

**Table 2:** Distribution of Disease-Modifying Therapies.

Medication	Patients (n)	% of Total Cohort
**Moderate Efficacy Disease-Modifying Therapies**		
Interferon-beta products	863	84.2%
Glatiramer acetate	107	10.4%
Dimethyl fumarate	41	4.0%
Teriflunomide	14	1.4%
**Total**	**1025**	**97.5%**
**High Efficacy Disease-Modifying Therapies**		
Fingolimod	15	1.4%
Natalizumab	8	0.8%
Ocrelizumab	3	0.3%
**Total**	**26**	**2.5%**

## Data Availability

Anonymized data will be shared after request of a qualified investigator.

## Abbreviations

The following abbreviations are used in this manuscript:

BREMSO	Bayesian Risk Estimate for MS at Onset
BRS	Barcellona Risk Score
CI	Confidence Intervals
CSF	Cerebrospinal fluid
DMT	Disease modifying therapy
EDSS	Expanded Disability Status Scale
HE DMTs	Moderate efficacy disease modifying therapies
HR	Hazard ratios
IQR	Interquartile range
ME DMTs	Moderate efficacy disease modifying therapies
MRI	Magnetic resonance imaging
MS	Multiple sclerosis
MSSS	Multiple Sclerosis Severity Score
OCB	Oligoclonal bands
RRMS	Relapsing remitting multiple sclerosis
RISM	Italian multiple sclerosis and related disorders register
RoAD	Risk of Ambulatory Disability
SD	Standard deviation
SPMS	Secondary Progressive Multiple Sclerosis
